# Human Ratings of Writing Quality Capture Features of Syntactic Variety and Transformation in Chinese EFL Learners’ Argumentative Writing

**DOI:** 10.3389/fpsyg.2021.660796

**Published:** 2021-11-17

**Authors:** Jin Xue, Liyan Zheng, Xiaoyi Tang, Banban Li, Esther Geva

**Affiliations:** ^1^School of Foreign Studies, University of Science and Technology Beijing, Beijing, China; ^2^Ontario Institute for Studies in Education, University of Toronto, Toronto, ON, Canada

**Keywords:** valid indices, Coh-Metrix, foreign language context, rating, qualitative measures

## Abstract

Traditionally, writing quality is measured by human ratings, either holistically or analytically. The present study aimed to investigate the locus of human ratings by analyzing the linguistic features that are predictive of writing quality. One hundred and 44 argumentative writing samples from Chinese learners of English as a foreign language were evaluated by human ratings and quantitative measurement of writing quality indexed by Coh-Metrix. Holistic and analytic human ratings had significant correlations with quantitative measures related to syntactic variety and transformation. Moreover, linear and logistic regressions revealed that syntactic simplicity, words before main verb, syntactic structure similarity in all sentences and across paragraphs, incidence of passive voice and temporal connectives were five valid indices that can consistently differentiate writing quality indexed by human ratings. The present findings have significant pedagogical implications for human ratings on writing quality in the foreign language learning context.

## Introduction

Human ratings are widely used in assessing writing quality in a variety of educational tests. Holistic and analytic ratings using a scoring rubric are two traditional methods. Holistic ratings evaluate writings according to overall quality or “sense of whole” (Rosenthal, 1984), while analytic ratings score multiple aspects in a writing task. Holistic ratings are efficient, especially when a composition requires higher-order thinking (Nilson, 2010). Analytic ratings show merits in qualifying multiple features (Klein et al., 1998), and thus give more diagnostic information for a writing sample (Johnson and Hamp-Lyons, 1995).

Although holistic and analytic ratings have high correlations (Bauer, 1981; Zhang et al., 2015) or high level of similarity with each other (Bacha, 2001; Zhang et al., 2015), previous research on writing in the first language (L1; Swartz et al., 1999; Nordquist, 2020) and the second language (L2; Zhang et al., 2015) reveals a lack of validity and reliability in both rating methods. It is argued that different raters are likely to focus on different aspects of the written product in holistic ratings, and the criteria might restrict their views on merits of the writing sample (Babin and Harrison, 1999). Previous studies have reported holistic human ratings are differentially related to different aspects of linguistic features. For example, holistic rating scores have a weak correlation with grammar errors, but a stronger relation with mechanics (Kyle et al., 2014). And holistic ratings do not provide detail feedback, so writers are unsure about the content and quality of the writing. To increase the reliability, a clearly written scoring rubric is suggested for different features in a writing task. For instance, one grade should be given to the content coverage and another grade to writing quality (Calfee and Miller, 2013). This approach aligns with analytic ratings, scoring different features of a writing sample including content, organization, vocabulary, grammar, cohesion, mechanics, etc. However, researchers have questioned the possibility for raters to score more than three features simultaneously in analytic ratings (Underhill, 1987). Moreover, the scores assigned by different raters and by different methods (holistic and analytic) are found different (Zhang et al., 2015). Specifically, higher scores were assigned under analytic scoring for participants with lower writing proficiency, but participants with higher proficiency received higher scores under holistic scoring. It seems that human ratings are susceptible to subjectivity.

However, little is known about how writing quality assessed by different types of human ratings (holistic vs. analytic) is related to linguistic features for Chinese natives who learned English as a foreign language (EFL). The present study tapped into what indices of linguistic features were predictive of different types of human ratings in argumentative writings for Chinese natives EFL learners. For one, argumentative writing is a genre dominant in various academic writings like term papers, journal articles and dissertations at college. For another, it is argued that EFL writers are characterized by distinct syntactic structures from English L1 or L2 learners (Nasseri, 2021). It is important to understand which features English-language ‘high-quality’ argumentative writing has in the EFL context. Research findings in the present study are expected to shed light on the locus of human ratings in the EFL context. To be specific, the present findings will provide evidence of the specific linguistic features that are predictive of holistic vs. analytic human ratings in the Chinese EFL context.

### Measurement of Linguistic Features

The present study measured linguistic features of writing samples in a series of indices of syntactic complexity, an important construct in writing research (Jagaiah et al., 2020). Syntactic complexity taps the full range of linguistic resources offered by the given grammar in order to fulfill various communicative goals successfully (Ortega, 2003, 2015). Namely, syntactic complexity is an expansion of the ability to use the language more maturely and skillfully.

Measurement of writing quality is traditionally operationalized in a variety of large-grained indices of syntactic complexity like mean length of linguistic units (e.g., sentence, T-unit, causes). However, measurement on T-unit (Minimum Terminable Unit) and error free T-unit (Casanave, 1994), if implemented by hand coding, was criticized for its subjectivity. Further, the traditional linguistic measures of syntactic complexity like T-unit and mean length of T-unit (MLTU) are parsimonious since they are prone to interpretation difficulty (Norris and Ortega, 2009) and the possibility of misplacing focus on clausal subordination (Biber et al., 2011). To address these challenges, recent studies have improvised finer-grained measures of syntax complexity by capturing sophistication and variety dimensions of linguistic features like amount of subordination, amount of coordination, and degree of phrasal sophistication (Norris and Ortega, 2009; Lu, 2010; Bi and Jiang, 2020). Fine-grained indices like subordination and phrasal density are valid in distinguishing English writing quality at different Common European Framework of Reference levels for EFL learners with different L1 backgrounds (Khushik and Huhta, 2020). Previous research reports measures in syntactic elaboration and diversity explained 45.3% of the variance in predicting writing scores of secondary school EFL learners in narration (Bi and Jiang, 2020). Accordingly, except the traditional large-grained indices of syntactic complexity like mean length of sentence, fine-grained indices such as amount of different syntactic structures (e.g., Ortega, 2003, 2015) and the degree of phrasal and clausal sophistication (Deng et al., 2020) were also included in the present study.

To elaborate, the present study conceptualizes syntactic complexity under the notion of variety and sophistication of grammatical resources exhibited in language production (Ortega, 2003; Lu, 2011; Bulté and Housen, 2014). Variety and sophistication, respectively, refer to the arrangement and the extent of complexity in syntactic structures (Crossley and McNamara, 2014). The variety dimension of syntactic complexity can be indexed by the degree of sentence simplicity and the density of syntactic transformation (e.g., the use of gerund, infinitives) etc., while typical indices for sophistication include the length of language output (e.g., mean length of sentence and mean length of clause, and clausal subordination), the density of complex or compound sentences (e.g., number of coordinate structures, number of subordinate structures), and the degree of phrase complexity (Crossley and McNamara, 2014), and syntactic embeddings (e.g., incidence score of different types of connectives).

### The Relationship Between Writing Quality and Syntactic Complexity in L2

A large body of research has addressed the relationship between writing quality and different measures of syntactic complexity in L2 (e.g., Beers and Nagy, 2009; Lu, 2011, 2017; Crossley and McNamara, 2014; Kyle and Crossley, 2018; Wu et al., 2020). Akin to L1 studies, studies on multiple writing corpora of different groups of learners at different time points have revealed a developmental pattern of syntactic complexity (Casanave, 1994; Lu, 2011; Bulté and Housen, 2014; Rosmawati, 2014). Development in indices of syntactic complexity is usually in line with writers’ proficiency level (Hwang et al., 2020; Atak and Saricaoglu, 2021). For example, Casanave (1994) tracked over three semesters the syntactic complexity of Japanese EFL writers. Results showed growth in the mean length of clauses, as well as in complex structures. In another study, using the average number of clauses per T-unit, Chinese EFL learner’s writing was found to become more grammatically complex over a six-month period (Larsen-Freeman, 2009). Norris and Ortega (2009) found L2 learners’ writing followed a developmental pattern of syntactic complexity from coordination at the beginning stage, to subordination at intermediate stage and to phrasal structures at the advanced stage. In a case study, Rosmawati (2014) explored complexity development in the academic writing of an advanced L2 learner during her postgraduate study in Australia over one academic semester. She found a significant increase in the uses of compound, complex, and compound-complex sentences over the year, and this increase was reflected in an overall improvement in her quality of writing in English. This line of empirical studies has provided evidence for the developmental stages of syntactic complexity hypothesized by Biber and colleagues (Staples et al., 2016).

Along the same line, a robust association is well established between measures of syntactic complexity and writing quality (e.g., Taguchi et al., 2013; Crossley and McNamara, 2014; Martínez, 2018). For instance, the correlations are significant between writing quality and indices of linguistic features for a writing sample like MLTU and occurrence of finite clausal subordination (Homburg, 1984). Thus, the indices of syntactic complexity like mean lengths of clause and complex nominals per clause are predictive of writing quality (Biber et al., 2016). Further, some measures of syntactic complexity are reliable in differentiating L2 writing quality. To specify, writings with high vs. low writing quality differed on several indices of syntactic complexity like mean length of sentence, MLTU, mean length of clause, clauses per T-unit, the amount of subordination and coordination, as well as the degree of phrasal complexity (Ortega, 2003, 2015). High-quality writing is characteristics of more complex phrases (e.g., complex nominal) and longer writing units (e.g., sentences, clauses, T-unit; Casal and Lee, 2019). Taguchi et al. (2013) analyzed a collection of argumentative essays written by non-native English speakers and found that noun phrase modification contributed to essay quality. This line of study supports indices of syntactic complexity reflect writing quality.

Similar findings were observed in writings by Chinese EFL learners. Research supports argumentative writing by EFL learners follows a developmental pattern “utilizing noun phrase complexity features to a greater extent over time” (Gray et al., 2019, p. 20). Relative to emerging writers, expert writers tended to use higher length of T-units, clauses, and sentences, and more usage of complex nominals, subordinate clauses and verb phrases in academic writing (Wu et al., 2020; Yin et al., 2021). An association between syntactic complexity and writing quality is well established for the Chinese EFL learners (Lu, 2010; Yang et al., 2015). Syntactic complexity as measured by mean length of sentences and MLTU correlated positively and significantly with writing quality (Yang et al., 2015). Quantitative analysis on college-level EFL writings reveals the correlation between human ratings and syntactic complexity scores indexed by length of production unit, amount of subordination, amount of coordination, degree of phrasal sophistication, overall sentence complexity ranges from 0.834 to 1.000 (Lu, 2010).

However, higher level of syntax complexity did not necessarily implicate higher writing quality. For instance, English writing by highly proficient native German speakers was more complex in terms of longer sentences, clauses, and T-units than those by native English speakers (Lu and Ai, 2015). Research articles by writers of English as a Lingua Francas have features of longer sentences, and greater reliance on nominal phrases, coordinate phrases and complex nominals compared to those by English natives (Wu et al., 2020). The contradictory findings are likely attributable to the transfer effect of L1 language properties. Previous research supports a positive correlation between language complexity in L1 and L2 writing (Ströbel et al., 2020). In the case of English writings by native German speakers, higher complexity in length of production unit coincides with the fact that German sentences tend to be longer than English sentences (Ziegler, 1991, p. 147).

Different L1 background (Lu and Ai, 2015; Ströbel et al., 2020) and genre (Yoon and Polio, 2017; Nasseri, 2021) have an effect on linguistic features of writings. Syntactic complexity in L2 writing is susceptible to the degree of syntactic complexity in L1 (Zenouzagh, 2020). So far, few studies are devoted to singling out syntactic complexity measures that can effectively contribute to writing quality indexed by different types of ratings in the Chinese EFL context. There is necessity to tap into the locus of human ratings by conceptualizing writing quality as a multi-dimensional construct and specifying finer linguistic features that can effectively account for L2 writing quality indexed by different types of human ratings.

## The Present Study

The present research aimed to investigate linguistic features that are predictive of writing quality indexed by holistic vs. analytic human ratings on Chinese EFL college-level argumentative writing. The following research questions were addressed: (1) What is the relationship between indices of linguistic features and writing quality measured by holistic and analytic human ratings? (2) What indices of linguistic features can validly distinguish holistic and analytic ratings for Chinese EFL argumentative writings?

To answer the above questions, the present study analyzed different dimensions of syntactic complexity and captured valid indices of syntactic complexities that can be used to differentiate high vs. low quality writing assessed by human ratings. To be specific, the present study used traditional large-grained indices of syntactic complexity (e.g., mean length of sentence, MLS) and fine-grained indices of syntactic complexity at phrasal, sentential and clausal levels to predict writing quality in argumentative writing. Writing quality in the present study was assessed by both holistic and analytic ratings by human raters. The traditional rating method, holistic scoring, is criticized for inadequacy in distinguishing linguistic features. The details in analytic rating enable fine judgment and thus boost the general impression in holistic rating, and analytic ratings are more likely to provide more diagnostic assessment on writing quality (Weigle, 2002). Analytic ratings improve the reliability and avoid bias between raters on the judgments on writing quality.

Given that manual calculation of indices of syntactic complexity is time, energy and expertise, researchers often opt for measures that are consistent in literature and efficient to calculate. With advancements of technology, in recent years, automatic quantitative analysis tools like Coh-Metrix allow a more fine-grained measurement of syntactic complexity (Kyle and Crossley, 2018) and thus are extensively used to derive indices characterizing linguistic features of syntax (Graesser et al., 2014). Thus, to capture linguistic features, the present study used Coh-Metrix 3.0 to derive measurement of syntactic complexities (for details, please see the method section). The use of Coh-Metrix allows for a number of syntactic complexity dimensions and their measures to be automated and examined. Following Crossley and McNamara (2014), we operationalized syntactic variety at four dimensions (phrase types, syntactic transformations, sentence variety, and syntactic simplicity) and syntactic sophistication at three more dimensions (phrase length, syntactic embeddings and overall syntactic simplicity; for details, please see the Materials and Methods section).

It was hypothesized that some indices of syntactic complexity would predict human rating scores on writing quality for the EFL learners under study. It was further hypothesized some measures of syntactic complexity could validly differentiate high- vs. low-quality writings assessed either by holistic or analytic human ratings.

## Materials And Methods

### Participants

The data for the present study were collected from 64 freshmen of General English Program and 80 sophomores of English Double Degree Program in a university in China. These participants, aged 19–20years, majored in Science and Technology and were tested at the second semester of the academic year. The selection of the participants from two different grades took into consideration differentiating writing quality as well as maximizing the varieties of EFL writing. Generally, the two groups of college students are of intermediate to high English proficiency. However, sophomore students are supposed have higher writing proficiency than freshmen, since the two groups took two different English programs. The freshmen in the General English Program received about 4-h classroom English instruction plus 2-h on-line English course per week. The main objective of English instruction was foster students’ comprehensive language awareness in use, as well as in fluency and accuracy through language competence learning and practice, to enrich vocabulary, to broaden horizon in using English. The sophomores in the English Double Degree program had finished General English Program and currently received more than 16h of English classroom instruction per week at the weekend or in the evening. English competence in reading, writing, speaking, listening and translation were further enhanced in this program.

### Corpus

EFL learners from the above-mentioned two different English programs were assigned an argumentative writing task entitled “Should a government be allowed to limit the number of children a family can have? The essays were written on computer after class. Students were free to use dictionaries or search references online. No time limit was imposed. Prior to analysis, the corpus was cleaned to ensure correct formatting and spelling. Features of the syntactic complexity were supposed to reflect the quality of argumentative writings in the foreign language contexts.

### Holistic and Analytic Human Ratings

Writing samples from the above corpus were scored by the second and third authors, who have a Master’s degree in English linguistics. They had learned English for over 12years and passed the highest English proficiency test in China, i.e., TEM 8 (Test for English majors, level 8). They evaluated the quality of writing samples on both holistic and analytic rating scales.

Scales in the holistic rating rubric ranged from 1 to 5: (1) Severe confusion or underdevelopment; Severe and persistent errors in sentence structures or word usages. (2) Insufficient supporting ideas; Inappropriate or unrelated examples, explanations, and/or detailed information; Obvious inappropriate word usages. (3) Uses of some developed explanations to support or illustrate an idea; adequately organized and developed; Sufficient but probably inconsistent syntactic and word usages. (4) Roughly well organized and developed with appropriate and adequate explanations, examples, and/or detailed information; showing facility in language use, diversity of syntax and vocabulary, although with minor errors. (5) Well organized and developed with clear and appropriate explanations, examples and/or detailed information; complex syntactic diversity and appropriate word selection.

The analytic rating rubric has five dimensions: grammar, lexicon, global organization, local organization and supporting ideas (adapted from Abbuhl, 2011). Each dimension is associated with a 1 to 8 scale with 1 indicating inadequacy or inaccuracy and 8 meaning good variety or full sophistication in each dimension. Take grammar as an example, 1=Use of simple sentence structure but with serious and frequent errors in morph-syntax. 8=Use of various complex constructions effectively and accurately although there might be rare errors. The total analytic score for each writing sample is the sum of the ratings on the five analytic dimensions.

Before the rating task, both raters were trained by the first author of this study on the use of the rating rubrics. During the rating, they were blind to the information of the specific grade levels of the participants. The two raters were required to score the writing samples holistically and analytically by referring to the two rating rubrics. Interrater reliability between the two raters in the study was strong. Pearson Correlations between the two expert raters on holistic and analytic ratings found significantly high coefficients (holistic rating score, *r*=0.822; analytic grammar, *r*=0.873, analytic lexicon, *r*=0.814; analytic global organization, *r*=0.821; analytic local organization, *r*=0.766; analytic supporting ideas, *r*=0.754; *ps*<0.01), indicating the two raters had high inter-rater congruence.

### Syntactic Complexity Indices Derived From Coh-Metrix

As reviewed above, seven dimensions of syntactic complexity covering variety and sophistication of syntactic structures were measured in the present study (Table 1).

**Table 1:** Seven dimensions of linguistic features derived by Coh-Metrix.

Levels	Label	Indices	Measures
Phrasal	**Phrase length**		
	WBMV	Words before main verb	Mean number of words before main verb
	MNP	Modifier per noun phrase	Mean number of modifiers per noun-phrase
	**Phrase types**		
	NP	Noun phrase density	Incidence of noun phrases
	AP	Adverbial phrase density	Incidence of adverbial phrase
	VP	Verb phrase density	Incidence of verb phrases
	PP	Preposition phrase density	Incidence of prepositional phrases
Sentential	**Overall sentence complexity**		
	MLS	Mean length of sentence	Mean number of words in sentences
	**Syntactic transformation**		
	Passive	Passive voice density	Incidence score of agentless passive voice forms
	Negation	Negation density	Incidence score of negation expressions
	Gerund	Gerund density	Incidence score of gerund
	Infinitive	Infinitive density	Incidence score of infinitive
Clausal	**Syntactic variety**		
	Synsimiad	Syntactic structure similarity in all adjacent sentences	Mean degree of sentence syntax similarity in all adjacent sentences
	Synsimiall	Syntactic structure similarity in all sentences and across paragraphs	Mean degree of syntax similarity of all combinations across paragraphs
	**Syntactic embeddings**		
	CC	Causal connectives	Incidence score of causal connectives
	LC	Logical connectives	Incidence score of logical connectives
	ACC	Adversative and contrastive connectives	Incidence score of adversative and contrastive connectives
	TC	Temporal connectives	Incidence score of temporal connectives
	AC	Additive connectives	Incidence score of additive connectives
	**Syntactic simplicity**		
	Synsimp	Syntactic simplicity	Z score of text easability

In line with previous syntactic complexity frameworks (Bulté and Housen, 2014), these measures were realized at clausal, sentential and phrasal levels. To specify, the phrasal level of syntactic complexity involves lexical profiles indexed by phrase length and types (Laufer and Nation, 1995; Baba, 2009; Crossley and McNamara, 2012), the sentential level involves overall sentence complexity indexed by mean length of sentence and syntactic transformation indexed by incidence score of syntactic structures like passive, negation, gerund and infinitive (Ortega, 2003), and the clausal level covers syntactic variety and syntactic embeddings indexed by syntax similarity, syntactic simplicity, connectives types and number of connectives (Lu, 2011).

The automatic quantitative analysis tool Coh-Metrix (Graesser et al., 2011, 2014) was used to derive the above indices. The rationale for adopting the Coh-Metrix as the analytic tool was twofold: (1) It is an automated measurement for syntactic complexity that is freely accessible through a Web-based interface. (2) There are 106 indices of the linguistic and discourse representations of texts in Coh-Metrix. Seven dimensions with 19 measures (Table 1) selected in the present study have been used to investigate syntactic complexity in L2 writing. They were reviewed in Ortega (2003), Lu (2011), and Crossley and McNamara (2014), demonstrating positive relationships between these indices and writing quality.

The following explains how we derived indices *via* Coh-Metrix.

1. *Phrase length*. Coh-Metrix computes number of words before main verb. It is assumed that the longer the phrases, the more complex sentence is. And the number of modifiers per noun phrase (left embeddedness and embeddedness of noun phrases) is another index with a higher value indicating a higher degree of embeddedness and syntactic complexity (Crossley and McNamara, 2014).
2. *Phrase types*. Coh-Metrix provides incidence scores of various types of phrases, including adverbial phrase (AP: related to incidence of adverbial phrase), “noun phrase (NP: related to density of propositions), verb phrase (VP: related to the number of clauses in a sentence), and prepositional phrase (PP: related to the number of phrases that provide adjectival and adverbial information)” (Crossley and McNamara, 2014, p. 70).
3. *Overall sentence complexity*. Coh-Metrix computes mean length of sentence. Sentences with more words are supposed to have more complex syntax and may be more difficult to process.
4. *Syntactic transformation*. This dimension is measured by the normalized incidences of occurrences of different syntactic structures (Crossley and McNamara, 2014): agentless passive voice forms, negation expressions, gerund and infinitives. Such transformations represent syntactic complexity beyond the use of basic form of verbs in sentences.
5. *Sentence variety*. This dimension reflects “the consistency and uniformity of the clausal, and part of speech constructions located in the text” (Crossley and McNamara, 2014, p. 70). This dimension is evaluated by two indices: syntactic similarity in all adjacent sentences and syntactic structure similarity in all sentences and across paragraphs. The former is similar structures in successive sentences in a span of an essay while the latter is similar structures in all pairs of sentences. The two indices are calculated, respectively, by the proportion of intersection tree nodes between all adjacent sentences, and between all sentences and across paragraphs. More uniform syntactic constructions result in less complex syntax. However, high-quality writings by advanced writers are characteristic of more complex syntax structures in discourse (e.g., Casanave, 1994; Lu, 2010; Yang et al., 2015).
6. *Syntactic embeddings*. It is calculated in Coh-Metrix by the Charniak parser. The indices are in the form of normalized incidence counts (Crossley and McNamara, 2014, p. 70). The present study used indices of connectives to represent different types of syntactic embedding: causal connectives, logical *connectives*, adversative and contrastive connectives, temporal connectives, and additive connectives. The connectives contribute to cohesion of writing.
7. *Syntactic simplicity.* It is measured by Z score of text easability, which was derived by Principal Component Scores based on the length of words and sentences within the text in Coh-Metrix. Syntactic simplicity provides information on the degree that the text uses more complex, unfamiliar syntactic structures. The index is based on the assumption that syntactically complex sentences tend to include embedded constituents and are often structurally dense (Graesser et al., 2014).

### Statistical Analyses

The following analyses were run by R programming. Pearson correlation analysis was applied to examine the relation patterns between human ratings and features of syntactic complexity. To explore whether the same set of indices of syntactic complexity would consistently contribute to writing quality, two sets of linear regression using the indices of syntactic complexity as independent variables to predict wring quality assessed by holistic and analytic ratings, respectively. To further explore how the indices of syntactic complexity can be used to differentiate human ratings, two sets of logistic regressions analyses (stepwise) were performed. In the logistic regressions, only the indices of syntactic complexity with significant predicting power in linear regression were entered into the models as independent variables, and wring quality assessed by holistic or analytic ratings as dependent variables, respectively.

## Results

Table 2 showed the descriptive data.

**Table 2:** Descriptive data of English as a foreign language argumentative writings for the present samples.

	Label	Mean	SE
Phrase length	WBMV	5.18	0.16
	MNP	0.88	0.01
Phrase types	NP	353.91	2.29
	AP	33.14	1.21
	VP	229.31	2.96
	PP	93.31	1.82
Overall sentence complexity	MLS	18.53	0.53
Syntactic transformation	Passive	7.5	0.52
	Negation	11.08	0.76
	Gerund	12.93	0.83
	Infinitive	23.26	0.94
Syntactic variety	Synsimiad	0.1	0
	Synsimiall	0.09	0
Syntactic embeddings	CC	29.43	1.17
	LC	44.8	1.3
	ACC	18.04	0.78
	TC	14.69	0.69
	AC	44.78	1.18
Syntactic simplicity	Synsimp	−0.29	0.07
Ratings	Holistic rating	3.57	0.05
	Analytic rating sum	29.5	0.38
	Grammar	5.01	0.08
	Lexicon	5.1	0.08
	Global organization	5.24	0.08
	Local organization	5.34	0.08
	Supporting ideas	5.22	0.08

**Figure 1:**
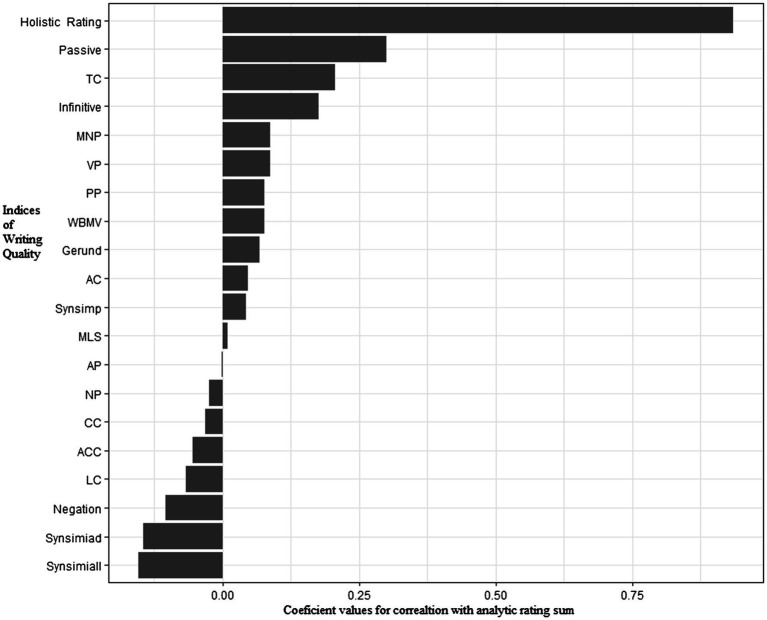
Patterns of correlations between syntactic complexity indices and analytic ratings.

**Figure 2:**
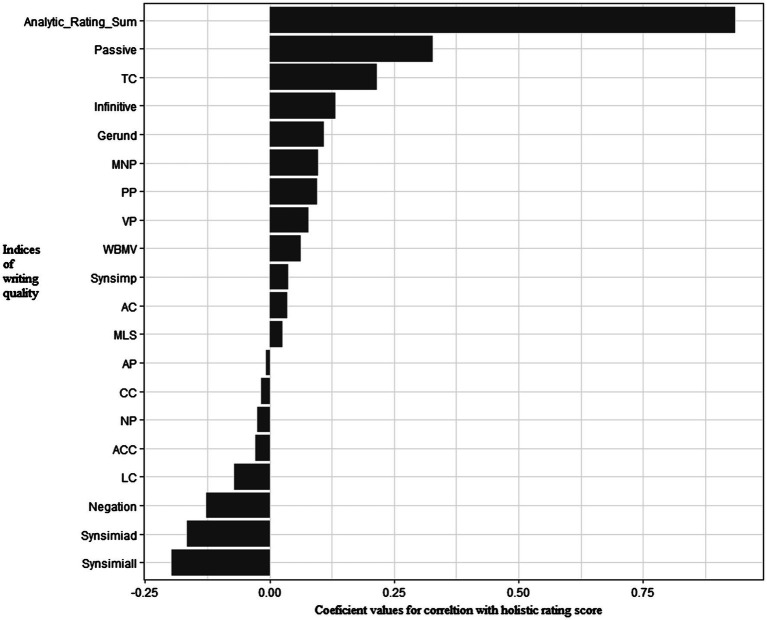
Patterns of correlations between syntactic complexity indices and holistic ratings.

Pearson correlation analysis showed that holistic rating was positively correlated with the sum score of analytic rating sum, *r*=0.094, *p*<0.001. Table 3 summarizes results of Pearson correlation analysis between indices of syntax complexity and human ratings across dimensions. Four Coh-Metrix indices (Synsimiad, Synsimiall, Passive, and TC) were significantly correlated with holistic ratings (*ps*<0.05) and 4 Coh-Metrix indices (Synsimiall, Passive, Infinitive, and TC) demonstrated significant correlations with analytic ratings (*ps*<0.06). Figures 1, 2 show patterns of correlations between indices of writing quality and two types of human ratings.

**Table 3:** Correlation between indices of linguistic features and human ratings.

	Holistic ratings		Analytic ratings sum		Grammar		Lexicon		Global organization		Local organization		Supporting detail	
	*r*	*p*	*r*	*p*	*r*	*p*	*r*	*p*	*r*	*p*	*r*	*p*	*r*	*p*
MLS	0.03	0.76	0.01	0.92	0.02	0.83	0.10	0.25	−0.10	0.25	−0.02	0.81	0.03	0.73
Synsimp	0.04	0.66	0.04	0.62	0.01	0.94	−0.08	0.36	0.12	0.15	0.08	0.34	0.04	0.62
WBMV	0.06	0.46	0.08	0.37	0.14	0.09	**0.19**	**0.02**	−0.07	0.37	0.03	0.74	0.04	0.67
MNP	0.10	0.25	0.09	0.30	**0.16**	**0.05**	**0.20**	**0.02**	−0.03	0.69	0.04	0.66	−0.01	0.87
NP	−0.02	0.78	−0.03	0.76	−0.01	0.92	0.11	0.20	−0.10	0.24	−0.04	0.65	−0.06	0.47
AP	−0.01	0.94	0.00	0.99	−0.04	0.65	−0.01	0.92	0.09	0.27	−0.08	0.37	0.03	0.75
VP	0.08	0.36	0.09	0.30	0.03	0.68	−0.11	0.19	0.15	0.07	**0.17**	**0.05**	0.12	0.17
PP	0.10	0.25	0.08	0.36	0.13	0.13	**0.19**	**0.02**	−0.07	0.39	0.02	0.84	0.04	0.62
Synsimiad	**−0.17**	**0.05**	−0.15	0.08	−0.08	0.32	**−0.24**	**0.001**	−0.09	0.29	−0.10	0.25	−0.08	0.36
Synsimiall	**−0.20**	**0.02**	**−0.15**	**0.06**	−0.10	0.22	**−0.23**	**0.01**	−0.08	0.37	−0.12	0.15	−0.08	0.31
Passive	**0.33**	**0.001**	**0.30**	**0.001**	**0.34**	**0.00**	0.10	0.22	**0.24**	**0.00**	**0.28**	**0.00**	**0.23**	**0.00**
Negation	−0.13	0.13	−0.10	0.22	−0.14	0.10	−0.11	0.20	−0.02	0.80	−0.12	0.17	−0.03	0.74
Gerund	0.11	0.19	0.07	0.42	**0.22**	**0.01**	0.16	0.06	−0.08	0.33	−0.01	0.92	−0.04	0.65
Infinitive	0.13	0.12	**0.17**	**0.04**	0.14	0.09	0.12	0.17	0.11	0.20	**0.22**	**0.01**	**0.16**	**0.05**
CC	−0.02	0.85	−0.03	0.71	−0.02	0.84	−0.01	0.91	−0.04	0.62	−0.03	0.75	−0.04	0.61
LC	−0.07	0.40	−0.07	0.42	−0.12	0.16	−0.03	0.75	−0.02	0.77	−0.09	0.31	−0.02	0.84
ACC	−0.03	0.73	−0.06	0.51	−0.11	0.19	−0.06	0.50	0.02	0.77	−0.05	0.58	−0.05	0.53
TC	**0.22**	**0.01**	**0.21**	**0.01**	**0.23**	**0.00**	0.11	0.21	**0.18**	**0.03**	**0.18**	**0.03**	0.13	0.13
AC	0.04	0.67	0.05	0.59	−0.02	0.82	0.05	0.58	0.02	0.83	0.03	0.69	0.12	0.16

The bold values means ps < 0.01.

The indices of syntactic complexity with significant correlations with the five aspects of analytic ratings generally coincided with those with Analytic Ratings Sum. Additionally, rating on grammar had significant correlations with MNP (*r*=0.16, *p*=0.05) and gerund (*r*=0.22, *p*=0.01); Rating on lexicon had significant correlations with WBMB (*r*=0.19, *p*=0.02), MNP (*r*=0.20, *p*=0.02), PP (*r*=0.19, *p*=0.02) and Synsimiad (*r*=−0.24, *p*=0.001). Accordingly, in the following multiple linear regression analysis, indices of syntactic complexity were used to predict the two major ratings (analytic vs. holistic) of writing quality.

In the following regressions, Beta weights were used in order to compare the contributions of each variables. Beta weights are the standardized regression coefficients, representing the slope of a line in a regression equation (Pedhazur, 1997). In the equation with multiple predictor variables, *β* can be larger than +1 or smaller than −1. This was determined by calculating the individual coefficient estimates and the corresponding standard error for each of the estimates.

Results of multiple regression analysis indicated in predicting analytic ratings, the indices explained 24% of the variance in predicting analytic ratings of writing quality, *R*^2^=0.24, *F* (19, 124)=2.11, *p*=0.008 (Table 4). Four syntactic indices were included as significant predictors of the analytic ratings: WBMV (*β*=0.58, *p*=0.03), Passive (*β*=0.14, *p*=0.06), Infinitive (*β*=0.08, *p*=0.07), and TC (*β*=0.11, *p*=0.018). In predicting the holistic ratings, the indices of syntactic complexity explained 27% of the variance, *R*^2^=0.27, *F* (19, 124)=2.43, *p*=0.002. It was found five syntactic indices predicted writing quality indexed by holistic ratings: Synsimp (Syntactic simplicity; *β*=0.26, *p*=0.096), WBMV (Words before main verb; *β*=0.07, *p*=0.06), Synsimiall (Syntactic structure similarity in all sentences and across paragraphs; *β*=−7.80, *p*=0.04), Passive (Passive voice density; *β*=0.02, *p*=0.03), and TC (Temporal connectives; *β*=0.02, *p*=0.01).

**Table 4:** Linear regression in predicting writing quality assessed by human ratings.

Dependent variables								
	Analytic rating				Holistic rating			
Predictors	*β*	SE	*t*	*p*	*β*	SE	*t*	*p*
(Intercept)	15.73	11.69	1.35	0.18	1.58	1.59	0.99	0.32
MLS	−0.04	0.13	−0.30	0.77	0.00	0.02	−0.17	0.86
Synsimp	1.72	1.16	1.49	0.14	0.26	0.16	1.68	**0.096** ^**#**^
WBMV	0.58	0.27	2.18	**0.03** ^*^	0.07	0.04	1.90	**0.06** ^**#**^
MNP	3.20	3.38	0.95	0.35	0.50	0.46	1.08	0.28
NP	0.02	0.02	0.87	0.38	0.00	0.00	0.89	0.37
AP	0.01	0.03	0.43	0.67	0.00	0.00	0.24	0.81
VP	0.00	0.02	0.21	0.83	0.00	0.00	0.45	0.65
PP	0.01	0.03	0.54	0.59	0.00	0.00	0.71	0.48
Synsimiad	−8.59	22.20	−0.39	0.70	0.67	3.02	0.22	0.82
Synsimiall	−34.57	28.00	−1.24	0.22	−7.80	3.81	−2.05	**0.04** ^*^
Passive	0.14	0.07	1.88	**0.06** ^**#**^	0.02	0.01	2.16	**0.03** ^*^
Negation	0.00	0.05	0.03	0.98	0.00	0.01	−0.09	0.93
Gerund	0.02	0.04	0.40	0.69	0.00	0.01	0.62	0.54
Infinitive	0.08	0.04	1.78	**0.07** ^**#**^	0.01	0.01	0.98	0.33
CC	−0.01	0.04	−0.33	0.74	0.00	0.01	0.24	0.81
LC	−0.01	0.04	−0.37	0.71	0.00	0.01	−0.63	0.53
ACC	−0.01	0.05	−0.30	0.76	0.00	0.01	0.29	0.77
TC	0.11	0.05	2.40	**0.018** ^*^	0.02	0.01	2.58	**0.01** ^*^
AC	0.04	0.03	1.45	0.15	0.01	0.00	1.35	0.18

* *p*<0.05;

# *p*<0.1. The bold values means ps < 0.05.

Two sets of logistic regressions were run to further examine to what extend those indices of syntactic complexity can differentiate writing quality, which was, respectively, indexed by two groupings: by analytic ratings, and by holistic Ratings. To avoid the possibility of differences in language proficiency ensued from sampling from two different grades, human rating scores were turned into standardized scores, Z-scores, based on the grade mean and scaled on values ranging from −4 to 4. Z scores provide a possibility to obtain an evaluation on two different samples on an equal perspective (McLeod, 2019). Writings were grouped into high- vs. low-quality, respectively, by a cutoff of ±0.5 based on Z-scores of analytic ratings (high-quality, *N*=46; low-quality, *N*=45) and holistic ratings (high-quality, *N*=68; low-quality, *N*=54). The six syntactic complexity indices with significant predictive power in the linear regression analysis (i.e., Synsimp+WBMV+Synsimiall+Passive+Infinitive+TC) were used to predict the likelihood of differentiating writing quality (high vs. low). Variables in the equations of the logistic regressions are reported in Table 5.

**Table 5:** Variables in the equations of the logistic regressions.

Dependent variables								
	Analytic rating				Holistic rating			
Predictors	*β*	SE	*z* value	Pr(>|z|)	*β*	SE	*z* value	Pr(>|z|)
(Intercept)	−2.03	1.43	−1.42	0.16	−0.37	1.24	−0.3	0.77
Synsimp	1.01	0.49	2.08	0.04^*^	0.96	0.43	2.26	0.02^*^
WBMV	0.39	0.16	2.43	0.01^*^	0.34	0.14	2.39	0.02^*^
Synsimiall	−24.51	10.63	−2.31	0.02^*^	−32.1	9.74	−3.3	0.001^***^
Passive	0.13	0.05	2.86	0.004^**^	0.1	0.04	2.79	0.005^**^
Infinitive	0.02	0.02	0.86	0.39	0.01	0.02	0.4	0.69
TC	0.08	0.03	2.4	0.02^*^	0.08	0.03	2.78	0.005^**^

* *p*<0.05;

** *p*<0.01;

*** *p*<0.001.

The test on the difference between the residual deviance for the model with predictors and the null model (i.e., the number of predictor variables in the model) revealed that in predicting writing quality grouped by total scores of analytic ratings, Chi-square=29.98, *df*=6, *p*<0.001, the model’s log likelihood=52.12 (Table 5). The variables that explained significant variance in the equation included Synsimp, WBMV, Synsimiall, Passive and TC. In differentiating holistic ratings, Chi-square=38.48, *df*=6, *p*<0.001, the model’s log likelihood=71.10. The same valid variables can be used in differentiating holistic rating: Synsimp, WBMV, Synsimiall, Passive and TC.

## Discussion

The present study aimed to identify linguistic features that can differentiate high from low quality writings measured by holistic and analytic human ratings in college-level argumentative writings by Chinese native leaners of English. To capture linguistic features, syntactic complexity was conceptualized as a multi-dimensional construct and measured across seven dimensions covering linguistic features at clausal, sentential and phrasal levels. The analysis has demonstrated that linguistic features of syntactic complexity related to phrase and structure variety are consistently predictive of human holistic and analytic ratings on argumentative writing at the college-level in the Chinese EFL context.

The present study demonstrated that Synsimiad (Syntactic structure similarity in all adjacent sentences), Synsimial (Syntactic structure similarity in all sentences and across paragraphs), Passive (Passive voice density), Infinitive (Infinitive density), and TC (Temporal connectives) were five valid indices of syntactic complexity that can consistently differentiate high- from low-quality writings in the EFL context. These indices well capture variety and transformation dimensions of syntactic complexity. Based on the correlation results, higher-quality Chinese EFL writing seems to have a feature of higher level of syntactic variety at the sentential and clausal levels (i.e., Synsimial, Synsimiad). At the phrasal level, they used more transformed words (i.e., passive and infinitive voice forms). Thus, the results provide evidence that more syntactic variety and transformation are key features of high-quality argumentative writings at college-level. This finding concerning syntactic variety extends previous findings which characterized the sophistication dimension of syntactic complexity as involving greater number of different words and more sophisticated word choices (e.g., McNamara et al., 2015).

The present findings are in line with previous research (Jiang et al., 2019), demonstrating that a broader range of incidences of different types of clauses and noun modifiers (e.g., prepositional phrases and adjectival relative clauses) is associated with higher writing quality. However, indices of syntactic complexity predictive of writing scores among Chinese university students are not completely identical to those reported by Crossley and McNamara’s (2014) study recruited participants from university-aged L2 writers in an intensive writing class who were immersed in the English environment. Descriptive essays were examined in their study. This study derived similar syntactic complexity indices from the computational tool Coh-Metrix including measurements of syntactic variety, syntactic transformations, syntactic embeddings, incidence of phrase types, and phrase length. It was found incidence of all clauses, infinitives, and “that” verb complements were significant in predicting human evaluation on L2 writing quality. Divergent findings indicate variations in writing topic influence the relationship between syntactic complexity and writing quality (Yang et al., 2015). It is equally important to identify writing topics, English programs, and language proficiency in addressing which sub-constructs are powerful in differentiating writing quality or in articulating the relationship between writing quality and syntactic complexity.

Further, the present study reveals a slight different pattern of linguistic features that are predictive of writing quality indexed by holistic vs. analytic ratings. According to the results of regression analysis, indices at the phrasal level like infinitive density were associated with different dimensions of analytic rating (Table 3), and thus became valid predictors of analytic rating of writing quality (Tables 4 and 5). Comparatively, scores of syntactic simplicity and syntactic structure similarity in all sentences and across paragraphs explained significant variances of holistic ratings. The results indicate syntactic features at the phrasal level are better predictors for writing quality indexed by human analytic ratings, while indices at clausal level are more likely predictive of holistic ratings. The dissociation between predictors for holistic and analytic ratings provides evidence that syntactic features signifying L2 writing quality in analytic ratings may not necessarily the same syntactic features that will assist them in receiving higher holistic rating scores.

One surprising finding is we did not find significant correlations between human ratings of writing quality and some syntactic features like mean length of sentences, incorporating words before the main verb, modifier per noun phrase, negation, verb phrase, prepositional phrase revealed in previous studies (Bulté and Housen, 2014; Crossley and McNamara, 2014; Casal and Lee, 2019; Wu et al., 2020). Argumentative writings by advanced EFL learners are characterized by linguistic features of adverbial clauses, attribute adjectives embedded in the noun phrases and prepositional phrases as adverbials (Atak and Saricaoglu, 2021). On the one hand, the divergent results might be attributed to different measures for the multi-dimensional construct of syntactic complexity. Different syntactic complexity constructs and measures used in the present study were different from the above studies, which will invite conflicting results. On the other, it is highly possible that relatively lower English proficiency of the present participants constraints the production of more complex syntactic structures in argumentative writing. Previous findings support a developmental pattern for linguistic features in writing development. For instance, L1 Chinese EFL learners support argumentative writings drew heavily on grammatical structures like noun modifiers at beginning stages and phrasal modifiers at advanced stages (Atak and Saricaoglu, 2021).

The above findings point to the issue of content validity of human rating in evaluating writing quality. Weigle (2002) proposed that in the process of assessing writing quality, the rating method and standards are more likely to influence the results of rating scores. Writing quality rating in the present study followed the practice in several studies (Bulté and Housen, 2014; Martínez, 2018), where writing quality is indexed by both holistic and analytic ratings. Further, analytic rating takes into consideration of grammar, lexicon, global organization, local organization and supporting ideas. The present results support fine-grained phrasal or clausal indices like word transformation capture features of writing quality beyond the traditional indices of syntactic complexity like mean length of sentence (Kyle and Crossley, 2018). Thus, it is suggested human ratings should take into account more dimensions of syntactic complexity when evaluating argumentative writing in the EFL context. Specifically, linguistic features reflecting syntactic variety and transformation should be implemented in the rubric of human rating. However, it is cautious that the relationship between linguistic patterns and writing quality might not be straightforward. Previous studies revealed that relative to English natives, L2 learner groups overused passive structures in English-language writing (Lu and Ai, 2015) and used longer sentences, and greater reliance on phrases (Wu et al., 2020). Thus, the indices of syntactic complexity predictive of writing quality might not implicate the more incidences or more complex of these indices, the higher writing quality. This should be verified by comparing writing samples between Chinese EFL and English natives.

The present findings have implications for the locus of human ratings on writing quality. Primarily, in the Chinese EFL context, the ideal sub-constructs for argumentative writing quality should include at least two dimensions: syntactic variety and transformation. The similarity in syntactic structures, and the occurrence of infinitive and passive are important indices for features of EFL writing at the college level. Secondly, the practitioners will benefit from the present evidence on how analytic vs. holistic ratings differ or resemble. In addition to the dimensions of syntactic structures in rating rubric, human rating should take into consideration other factors, such as language proficiency, English programs and language context.

## Conclusion

In the present study, we have tried to circumvent the limitations of previous studies by conceptualizing writing quality as a multi-dimensional construct and measured it at multiple levels (the phrasal, sentential and clausal levels). Quantitative evidence as to the relationship between different dimensions of linguistic features and L2 writing quality was provided: First, writing quality assessed by both holistic and analytic human ratings had significant correlations with syntactic complexity measures related to syntactic variety and transformation. Second, syntactic simplicity, words before main verb, syntactic structure similarity in all sentences and across paragraphs, incidence of passive voice and temporal connectives were five valid indices of syntactic complexity that can consistently differentiate writing quality indexed by human ratings. Despite the findings, future studies should replicate the findings in the present study using longitudinal methods of data collection instead of samples from different English programs. In addition, different topics and tasks of writing can be used to validate the present findings.

## Data Availability Statement

The raw data supporting the conclusions of this article will be made available by the authors, without undue reservation.

## Ethics Statement

The studies involving human participants were reviewed and approved by the Human Subjects Review Board of University of Science and Technology Beijing. The patients/participants provided their written informed consent to participate in this study.

## Author Contributions

JX: research design, final draft writing, and data analysis. LZ: human ratings and first draft writing. XT: human ratings. BL: data preparation. EG: research design and comments and revision on the draft. All authors contributed to the article and approved the submitted version.

## Funding

The present research was supported by a grant from Social Science Foundation of Beijing, China (19YYB008).

## Conflict of Interest

The authors declare that the research was conducted in the absence of any commercial or financial relationships that could be construed as a potential conflict of interest.

## Publisher’s Note

All claims expressed in this article are solely those of the authors and do not necessarily represent those of their affiliated organizations, or those of the publisher, the editors and the reviewers. Any product that may be evaluated in this article, or claim that may be made by its manufacturer, is not guaranteed or endorsed by the publisher.
